# Evaluation of seasonal variation and the optimization of reducing sugar extraction from *Ulva prolifera* biomass using thermochemical method

**DOI:** 10.1007/s11356-021-12609-2

**Published:** 2021-02-05

**Authors:** Niyam Dave, Thivaharan Varadavenkatesan, Ram Sharan Singh, Balendu Shekher Giri, Raja Selvaraj, Ramesh Vinayagam

**Affiliations:** 1 Department of Biotechnology, Manipal Institute of Technology, Manipal Academy of Higher Education, Manipal, Karnataka 576104 India; 2 Department of Chemical Engineering and Technology, Indian Institute of Technology (IIT-BHU), Varanasi, Uttar Pradesh 221005 India; 3 Present Address: The Centre for Energy and Environmental Sustainability, Lucknow, 226001 Uttar Pradesh India; 4 Department of Chemical Engineering, Manipal Institute of Technology, Manipal Academy of Higher Education, Manipal, Karnataka 576104 India

**Keywords:** Green macroalgae, Structural carbohydrates, Periodic trend, Sugar profile, Thermal-acid hydrolysis, Characterization techniques

## Abstract

Green macroalgae comprise significant amount of structural carbohydrates for their conversion to liquid biofuels. However, it generally relies on species characteristics and the variability in seasonal profile to determine its route for bioprocessing. Hence, this study was conducted to analyze the indigenous marine macroalgal strain (*Ulva prolifera*) with respect to periodic trend and reducing sugar extraction. Consequently, in our investigation, the monthly variation in sugar profile and bioethanol yield was assessed between the monsoon and post-monsoon seasons, of which relatively high reducing sugar and fermentative bioethanol yield of about 0.152 ± 0.009 g/gdw and 6.275 ± 0.161 g/L was obtained for the October-month isolate (MITM10). Thereafter, the biochemical profile of this collected biomass (MITM10) revealed carbohydrate 34.98 ± 3.30%, protein 12.45 ± 0.49%, and lipid 1.93 ± 0.07%, respectively, on dry weight basis. Of these, the total carbohydrate fraction yielded the maximum reducing sugar of 0.156 ± 0.005 g/gdw under optimal conditions (11.07% (w/v) dosage, 0.9 M H_2_SO_4_, 121°C for 50 min) for thermal-acid hydrolysis. Furthermore, the elimination of polysaccharides was confirmed using the characterization techniques scanning electron microscopy (SEM) and Fourier transform infrared (FT-IR) spectroscopy. Therefore, the present thermochemical treatment method provides a species-specific novel strategy to breakdown the macroalgal cell wall polysaccharides that enhances sugar extraction for its utilization as an efficient bioenergy resource.

**Supplementary Information:**

The online version contains supplementary material available at 10.1007/s11356-021-12609-2.

## Introduction

In recent times, algae are projected as a potential and sustainable green resource for bioprocessing applications due to its fast growth rate, significant biomass yield, and exclusive biochemical composition (Suganya et al. 2016). According to their geo-distribution, they are generally considered as major aquatic flora that cover diverse group of photosynthetic eukaryotic organisms, which vary according to size, shape, habitat, and reproductive structures. Morphologically, like higher plants, algae contain rigid cell wall, nucleus and chloroplast. However, they are mainly cryptogamous and possess complex morphology that is divided into two groups based on taxonomy (Suganya et al. 2016; Manoylov 2014), viz., unicellular microalgae (e.g., dinoflagellates and diatoms) and multicellular macroalgae (e.g., riverine, estuarine, and marine algae). Among them, the marine macroalgal strains are further classified based on morphological characteristics as green (*Chlorophyta*), brown (*Phaeophyta*), and red (*Rhodophyta*) algae, which are abundantly found in coastal ecosystem (Ramachandra and Hebbale 2020). Of these, the green macroalgal strains (for example, *Chaetomorpha* sp., *Caulerpa* sp., and *Ulva* sp.) are mostly cosmopolitan in nature that constitute an essential part of marine food chain and bear endo-symbiotic microorganisms (Singh and Reddy 2014; Wichard et al. 2015). Broadly, in the context of marine biodiversity, the group of green macroalgae is dominated by the genus *Ulva* that belongs to the family *Ulvaceae* (Wichard et al. 2015). It mainly comprises the following intertidal marine macroalgal strains: *Ulva lactuca*, *Ulva rigida*, *Ulva intestinalis*, *Ulva fasciata*, *Ulva compressa*, *Ulva linza*, and *Ulva prolifera*. Physiologically, each of these eutrophic strains is stress-dependent with respect to its morphology and ubiquitously distributed in the coastline region. These strains are identified using morphology-based taxonomical approach (Palanisamy and Yadav 2017).

Structurally, the cell wall of green macroalgal species belonging to *Ulva* genus mainly consists of four polysaccharides, viz., cellulose, glucuronan, xyloglucan, and ulvan, which cumulatively comprise about 45% of biomass dry weight (Kidgell et al. 2019). Additionally, it also contains the storage polysaccharide (starch) surrounding the pyrenoid rings in the chloroplast structure (Kidgell et al. 2019). Of these, the substantial amount of cellulose content (Trivedi et al. 2013) in *Ulva* species can be utilized for the liquid biofuel production using different methods, namely steam-explosion, hydrothermal-liquefaction, microwave-irradiation, enzymatic saccharification, and thermochemical extraction using dilute acid/alkali or hydrogen peroxide treatment (Maneein et al. 2018). Between them, the acid-catalyzed thermochemical method (thermal-acid hydrolysis) is mainly used for the direct extraction of monosaccharides from *Ulva* spp. (Maneein et al. 2018) due to its treatment efficiency, economic viability, and eco-friendly approach, which results in the production of sugars (Yuan et al. 2018). For this, response surface methodology (RSM)-based mathematical models are commonly used for the optimization of the monomeric sugar extraction (Hii et al. 2015). This involves three major steps: performing the experiments, regression analysis, and model validation to determine process efficiency as well as interaction among the experimental variables. On the other hand, the diversity of these monosaccharides is directly related to the eco-physiological parameters and seasonal-effect (Dave et al. 2019; Qarri and Israel 2020). The monomeric sugar profile of *Ulva* is mainly composed of D-glucose, D-galactose, D-xylose, D-mannose, D-arabinose, L-rhamnose, and D-uronic acid (Yuan et al. 2018). Consequently, it is necessary to understand the biochemical composition and total reducing sugar profile of *Ulva* with respect to locality and the prevailing environmental conditions (Robin et al. 2017). Accordingly, an appropriate sampling strategy to find the most abundant locally available macroalgal strain is prerequisite for its conversion. In this milieu, for the first time, the present study focusses on the identification and characterization of the marine macroalgal strain (*Ulva prolifera*) obtained from the Malpe coastline of India, followed by optimization of reducing sugar extraction. The main objectives of this research include the following: (1) taxonomical identification and characterization of the *Ulva prolifera* biomass in terms of seasonal-effect as well as proximate components, (2) optimization of thermal-acid hydrolysis (TAH) in terms of biomass dosage, acid concentration, and hydrolysis time using RSM to improve the reducing sugar extraction.

## Materials and methods

### Reagents

For the seasonality study, proximate analysis, and reducing sugar estimation, all the chemicals were procured from Merck (India), HiMedia (India), and Loba Chemie (India). The reagents were of analytical grade and used without any modifications for the experimental studies.

### Collection and taxonomical identification of marine macroalgae

The macroalgal biomass was collected during monsoon and post-monsoon seasons (August to November, 2018) from Malpe beach (N13° 34′ 81.23″, E74° 69′ 58.42″), i.e., located in Udupi district of Karnataka on the southwest part of India. For the preliminary fieldwork, the quadrat sampling technique (Saito and Atobe 1970) was used for the biodiversity assessment using 50 × 50 cm iron grid, which is further divided into 25 sub-squares (10 × 10 cm each). The quadrat was placed in the field location and the occurrence of species was noted in each of the 25 sub-squares to determine frequency and coverage of the dominant macroalgal strain. After field sampling, the biomass collection was carried out using manual harvesting and according to the local tidal chart for the fieldwork. The collected biomass was washed with seawater to remove unwanted debris and stored in sterile plastic bags. The individual thalli were separated for morphological analysis as per the identification keys (Jha et al. 2009) and the field observation data. The macroalgal specimen was identified based on morphological characteristics (dark green coloration with branched and filamentous thalli) up to genus level by Energy and Wetlands Research Group, Centre for Ecological Sciences (IISc, Bengaluru, India). This was further sent for species authentication at Botanical Survey of India (Southern region, Coimbatore). Additionally, the wet preservation of the identified strain was also maintained at the laboratory conditions using 4% formaldehyde-fixative and sterile water for microscopic examinations.

### Pre-processing of biomass

Post-identification, the collected biomass was segregated, washed thrice with water (pH 7.2–7.3) in order to maintain the slight alkalinity near the pH value of the local seawater (7.5–8.4) (Tenjing et al. 2017) to avoid desiccation stress, and for the removal of excess salt, unwanted debris like sand particles, crustacean shells, or other marine epiphytes. Consequently, the washed biomass was dried in hot air oven at 70°C for 48 h until constant weight was obtained (Korzen et al. 2015). Furthermore, the biomass was ground into fine powder and sieved between BSS −18/+36 mesh size to improve the surface-to-volume ratio. Finally, the pre-processed sample was stored at ambient temperature in a sealed plastic container to avoid direct exposure to atmospheric moisture until further use.

### Seasonality studies

As part of the preliminary experimentation, the indigenous macroalgal biomass (*U. prolifera*) was coded as MITM8–MITM11 (Manipal Institute of Technology, collection area; Malpe, sampling code: 8-11) with respect to monthly collection. Subsequently, the hydrolysis experiments were conducted with 10% weight by volume (w/v) of each pre-weighed dried biomass using 1 mol/L (M) H_2_SO_4_ (Nunraksa et al. 2019) at 121°C for 60 min (Ra et al. 2013) in the 100-mL screw-cap glass bottles. For thermal acidolysis, the reaction mixtures in capped bottles were placed in a benchtop autoclave (7407PAD, Equitron Medica, India) to identify the seasonal trend of sugar profile and determine a suitable harvesting period. Then, the samples were neutralized and clarified, and the obtained centrate was used for the analysis of total sugar along with reducing sugar content as per phenol-sulfuric acid (DuBois et al. 1956) and 3, 5-dinitrosalicylic acid (DNS) method (Miller 1959). Apart from it, the total furfural derivative (e.g., TFA, total furanic aldehyde) in the hydrolysate was also estimated using chloroform extraction-based UV-detection method (Melo et al. 2016). After that, the bioethanol fermentation was carried out with conventional yeast (*Saccharomyces cerevisiae*) at 30°C, 150 rpm for 72 h in an orbital shaker (Kamath et al. 2018). Finally, the obtained bioethanol yield was analyzed using potassium dichromate method (Caputi et al. 1968; Althuri et al. 2017). For quantification, 1-mL aliquot of the samples were mixed with 4 mL of the 4% (w/v) potassium dichromate reagent, heated in a water bath at 60°C for 15 min, and rapidly cooled down to room temperature. Thereafter, the concentration of fermentative bioethanol in the broths were estimated by using a UV-Vis spectrophotometer (UV1800, Shimadzu, Japan) at 600 nm, using ethanol as the standard for normalization. On account of calculation, the sugar profile was quantified as amount of sugar content obtained in gram per gram dry weight of biomass (g/gdw) and the bioethanol yield was calculated against an internal calibration plot of known samples by comparing the empirically determined concentration of ethanol standard with respect to volumetric mass productivity in gram per liter (g/L) by assuming a maximum theoretical bioconversion yield of glucose to bioethanol of about 51%. Furthermore, to detect the maximum bioethanol production in the fermented broth, the gas chromatography (GC Clarus 590, Perkin Elmer, USA) was used under optimal condition using the COL-ELITE-2560 (cross bond-5% diphenyl-95% dimethyl polysiloxane) column (100 m × 0.25 mm ID) at 120°C, flame ionization detector (FID) at 250 °C, and injector at 250°C along with helium as carrier gas. Overall, the seasonal analyses were conducted in triplicates and the each biological isolate with reference to high biomass yield (green macroalgae) was repeated monthly wise in this study.

### Determination of biochemical composition

The proximate compositional analysis of the selected macroalgal biomass (MITM10) was conducted after the pre-processing steps for *U. prolifera* as per the standard procedures. For determining the biochemical components, all the experiments were performed in triplicates and the results are presented as mean ± standard deviation.

#### Carbohydrate analysis

For the total carbohydrate quantification, 60 mg of pre-processed macroalgal biomass was mixed with 2 mL of 2.5 M HCl solution and hydrolyzed at 95°C in water bath for 2 h. Then, the lysed sample was cooled down to ambient temperature and neutralized with 1 M NaOH. The mixture was centrifuged (CPR24 Plus, Remi, India) at 10,000 rpm for 10 min at 20°C and the obtained supernatant was used to estimate the total carbohydrate content using Dubois method (DuBois et al. 1956), wherein glucose was used as the standard.

#### Protein analysis

For the protein estimation, 100 mg of pre-processed macroalgal biomass was immersed in 4 mL of 1 M NaOH solution and protein extraction was carried out at 80°C for 1 h using water bath. After this, the extracted protein sample was cooled down to ambient temperature and allowed to settle for 5 min to separate the bottom layer. Then, the upper aqueous layer was separated and an aliquot of 100 μL was mixed with 3 mL of Bradford reagent for protein quantification (Bradford 1976), wherein bovine serum albumin was used as the standard.

### Lipid analysis

The lipid extraction was performed according to Bligh and Dyer method (Bligh and Dyer 1959) with slight modifications as suggested for *Ulva* sp. (Suthar et al. 2019). For the lipid analysis, 200 mg of pre-processed macroalgal biomass was suspended in 2 mL of chloroform: methanol mixture (2:1 v/v), vortexed and sonicated in ultrasonic-bath (XUBA1, Grant, Wolf Laboratories, UK) for 30 min followed by incubation at 25°C for 15 min. The resultant mixture was centrifuged at 10,000 rpm for 20 min at 20°C. Then, the clear supernatant was collected separately in a 15-mL polypropylene tube and the remaining pellet was resuspended in equal volume of the extractant for the residual lipid extraction following the same steps. After this, the obtained supernatant was mixed with 3.2 mL of distilled water and vortexed for 5 min. The tube was then kept for 4 h at room temperature for phase separation. The bottom layer and interphase were collected in a pre-weighed 1.5-mL Eppendorf tube and kept for drying in hot air oven at 60°C for 24 h until a constant weight was attained. After drying, the lipid content was estimated using gravimetric method.

#### Ash content analysis

About 3 g of the pre-processed macroalgal biomass was weighed using analytical balance (BSA822, Sartorius, Germany), transferred to a dry crucible, and placed in a muffle furnace at 550°C for 16 h (Liu 2019). The obtained ash content was determined with respect to weight difference using gravimetric method.

### Thermochemical treatment

#### Preparation of macroalgal hydrolysate

A predetermined quantity of dried macroalgal biomass was hydrolyzed with dilute sulfuric acid under the controlled process conditions (121°C, 15 psi) using an autoclave for a specific duration to release reducing sugar. The hydrolyzed sample was cooled to ambient condition. Then, the residual biomass slurry was separated by centrifugation at 10,000 rpm for 20 min at 20°C and the obtained acidic centrate was neutralized to pH 7 using 1 M NaOH. The resultant sugar extract containing sodium sulfate precipitate was centrifuged. Finally, the acquired clear supernatant was used for reducing sugar estimation.

#### Reducing sugar estimation

The reducing sugar content in the hydrolyzed sample was quantified using DNS method (Miller 1959). For analysis, 2 mL of the diluted sample was mixed with an equal volume of the DNS reagent, heated in a water bath at 90°C for 10 min, and instantly cooled down to room temperature. Then, the total amount of reducing sugar present in the macroalgal hydrolysate was measured by using UV-Vis spectrophotometer at 540 nm, using glucose as the standard. The reducing sugar yield (RSY) was calculated as the total amount of extracted reducing sugar in terms of g/gdw.

#### Experimental design

The preliminary screening experiments were conducted using one-factor-at-a-time approach to screen a host of factors for TAH of the selected macroalgal biomass (MITM10). Furthermore, the experiments were carried out using a central composite design (CCD) (Box and Wilson 1951) for the TAH optimization using the following screened factors: (A) biomass dosage (5–15% w/v), (B) sulfuric acid concentration (0.3–1 M), and (C) hydrolysis time (20–50 min). The low (−1), mid (0), and high (+1) values of each factor are represented by uncoded units, viz., A (5, 10, 15% w/v), B (0.3, 0.65, 1.0 M), and C (20, 35, 50 min), respectively (Table 2). The additional star points (−2 and +2) are denoted as A (1.59, 18.41% w/v), B (0.06, 1.24 M), and C (9.77, 60.23 min) in the matrix. Overall, the design includes eight corner points, six axial points, and six center points in the experimental setup, as generated by the software package Design Expert 10.0.2 (Stat Ease Inc., USA). Accordingly, in our investigation, RSM based on the three-level complete factorial design was applied as a method to investigate the individual and interaction effects on the reducing sugar extraction. Each process variable was evaluated at five coded levels (−2, −1, 0, +1, and +2) using full factorial design in one block, together with two star points and six center points. The coded and uncoded units in the CCD matrix included twenty experimental runs for TAH optimization. Based on statistically designed TAH experimentation, the optimum level of each significant independent variable was determined with RSY as the response. The significance of the obtained model and specific statistical parameters was assessed using analysis of variance (ANOVA). Moreover, the correlation between the response and the experimental level of each factor was represented in the form of 3-D surface plots. Along with this, the experimental and predicted values of RSY are shown in Table 2.

### Morphological and functional group characterization

The physical structure of the biomass before and after TAH was observed using a scanning electron microscope (EVO MA18, Zeiss, Germany). For topological analysis, the dried samples were mounted using a conductive tape and the images were captured at a magnification of × 10,000. Furthermore, the major modification in structural groups of the macroalgal biomass before and after TAH was analyzed using Fourier transform infrared (FT-IR) spectroscopy. The spectrum of the macroalgal biomass was obtained on a FT-IR spectrophotometer (8400S, Shimadzu, Japan) using potassium bromide pellets and recorded in the transmission mode in the range of 4000–400 cm^−1^.

## Results and discussion

### Identification of marine macroalgae

**Fig. 1:**
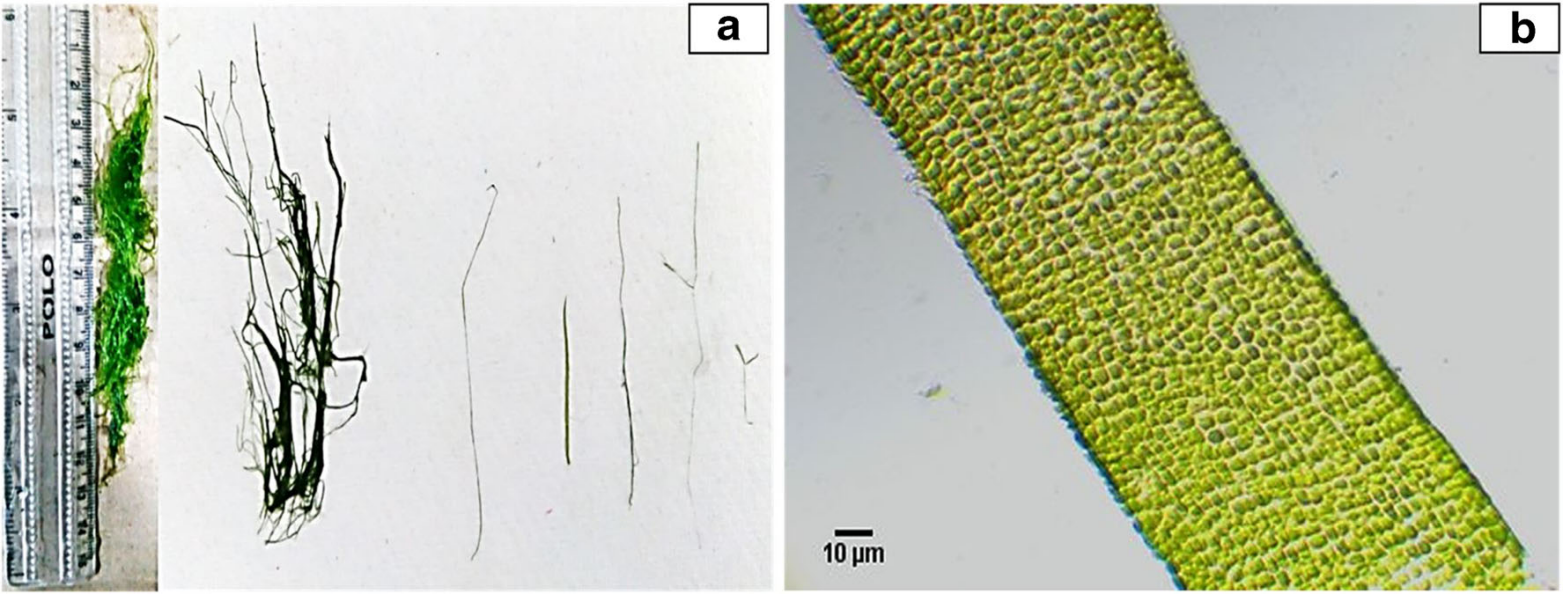
Taxonomical identification of marine macroalgae *Ulva prolifera* O.F. Muell. MITM. **a** Herbarium preparation. **b** Cross-sectional analysis under × 20 objective

Genus-level identification of the specimen led to the finding that it belonged to the *Ulva* species. The macroalgal strain was taxonomically verified as *Ulva prolifera* O.F. Muell. (Müller 1782) using specific identification keys (Guiry et al. 2014; Zhang et al. 2016) and the specimen was stored in the form of herbarium (Fig. 1a) at Department of Chemical Engineering, Manipal Institute of Technology, Manipal, India. According to the macroalgal biodiversity assessment conducted by CMFRI (India), there are about 78 species of marine macroalgae belonging to 52 genera and 28 families in the coastal region of Karnataka (Kaladharan et al. 2011). Of these, mainly one macroalgal strain (*U. prolifera*) was found to be abundant in nearby locality during monsoon and post-monsoon seasons, which we have collected in our present study. Furthermore, this isolated strain was named as “*Ulva prolifera* O.F. Muell. MITM.”

The green macroalgal strain, *Ulva prolifera* O.F. Muell. (also called as *Ulva prolifera* O.F. Müller) is the most dominant eutrophic species of marine algae, which is responsible for “green-tides.” It is classified under the phylum *Chlorophyta* and class *Ulvophyceae*, which belongs to the family *Ulvaceae*. As per the Fritsch’s taxonomy, this strain is categorized as a type species in the genus *Ulva* (previously known as *Enteromorpha*) that is globally distributed in various coastal environments and grows about 8–10 m above the sea level in its common habitat like rocky shores or intertidal areas. The frequency of this macroalgal strain is normally seasonal, i.e., high in monsoon and post-monsoon season, depending on several ecological parameters (e.g., salinity, temperature, light intensity, and nutrient concentration) that determines its plasticity and morphological characteristics. For example, the field studies conducted on the distribution of *U. prolifera* biomass in the Southern Yellow sea of China suggested that organic nitrogen and phosphorus in seawater promote its growth and biomass production (Li et al. 2016). Apart from this, the parenchymatous thallus of *U. prolifera* also shows tolerance to high salinity and desiccation stress (Gao et al. 2016) that indirectly provides an advantage comparative to other eutrophic strains of marine macroalgae, viz., *U. intestinalis* and *U. compressa*.

In general, the three *Ulva* spp. are closely related and commonly regarded as ambiguous species in phylogenetic analysis. However, these species are taxonomically segregated based on morphological features: macroscopic and microscopic. Among them, the macroscopic features include branching pattern and arrangement of thallus structure, for example, *U. intestinalis* encompasses unbranched hollow tubular thallus, whereas *U. compressa* comprises highly branched compressed thallus (Gao et al. 2016). On the other hand, *U. prolifera* shows some specific attributes (Zhang et al. 2016); it consists of light to dark green-colored branched thalli arising from a small discoid base; thallus is tubular and elongate and increases in width from base to mid thallus (Fig. 1a). Additionally, the microscopic features also include distinct cellular patterns; like in *U. compressa*, the cells are organized longitudinally forming rosette structure (Bast et al. 2014). *U. intestinalis* exhibits loosely stacked cells with light green coloration in contrast to *U. prolifera* (Zhang et al. 2016). Therefore, in the present study, post the identification using macroscopic features, the macroalgal specimen was also microscopically verified in the laboratory using a Euromex trinocular microscope under × 20 objective (Fig. 1b). The unique structural characteristics of *U. prolifera* were confirmed. Henceforth, the identified strain was documented using image processing software (ImageJ 1.52a, National Institute of Health, USA) and used for the seasonal variation experiments, biomass characterization, and TAH optimization studies.

### Biomass pre-processing

Pre-processing is usually defined as the preparatory phase, which tends to improve the biomass conversion efficiency prior to TAH process. Pre-processing normally includes methods like washing, oven drying, sun drying, or shade drying along with size reduction by various mechanical means for making the biomass suitable for both compositional analysis and TAH process. The washing step is to eliminate contaminants as well as excessive salinity and nutrients from seawater. The drying and size-reduction steps are carried out to meet the quality requirement (constant biomass weight and uniform particle size) for TAH process. Accordingly, the pre-processing steps for the collected biomass were performed. The *U. prolifera* biomass, when washed with water and oven dried, resulted in 10–15% dry weight under optimized conditions. Furthermore, the size reduction step yielded an average particle size of ≈ 400 μm.

### Analysis of seasonal effect

**Fig. 2:**
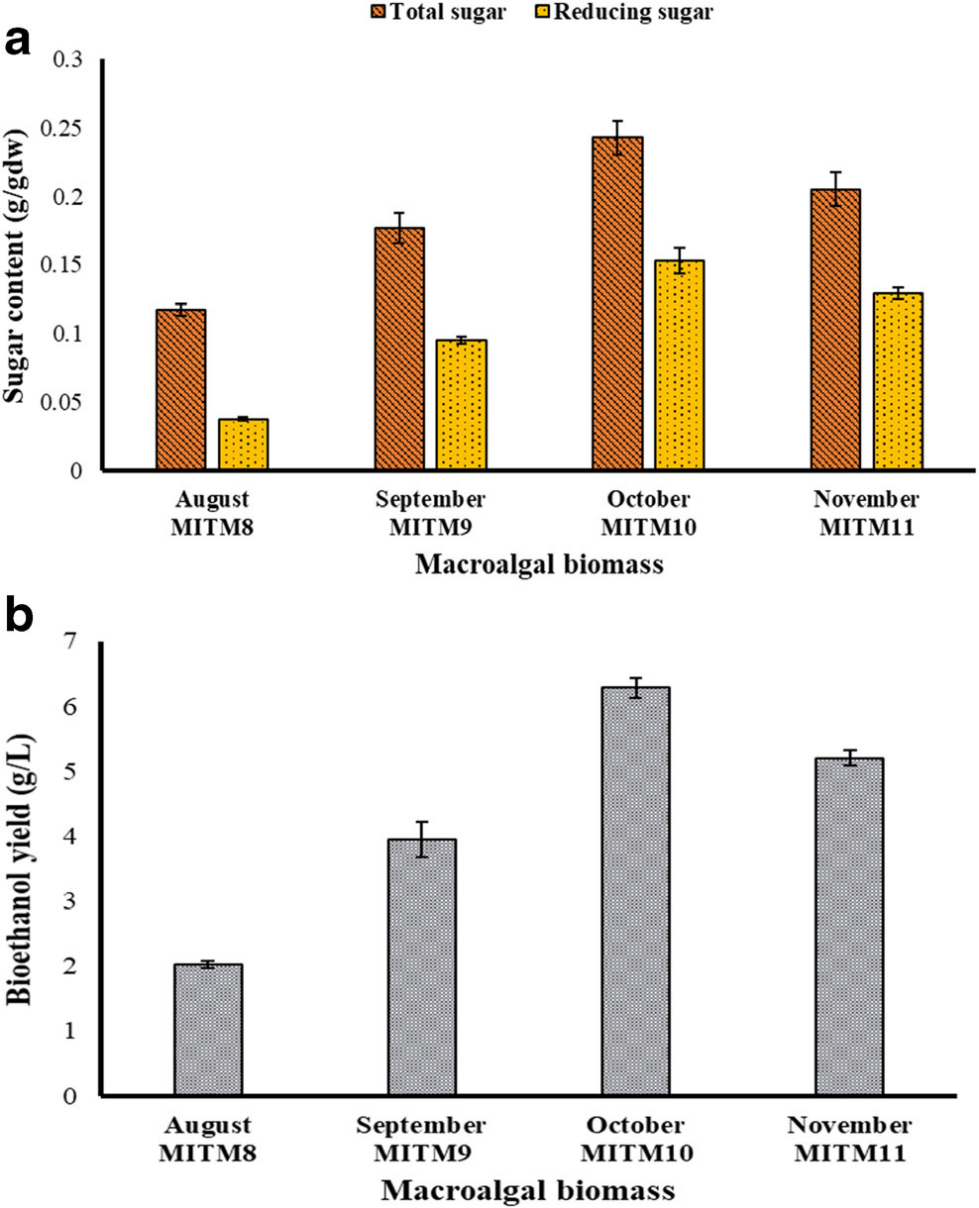
Evaluation of seasonal variation (August–November 2018) among the marine macroalgal specimens in terms of biochemical alterations. **a** Sugar content. **b** Bioethanol yield

The biochemical contents of marine macroalgae changes with seasonal fluctuations and attains its maxima at certain point under the suitable environmental conditions for a particular species, which determine its growth characteristics and appropriate harvesting period (Schiener et al. 2015). Thus, in this study, the sampling of the macroalgal species (*U. prolifera*) over the two different seasons (monsoon and post-monsoon) was carried out to analyze the alterations in biomass composition and its effect on subsequent bioethanol production (Fig. 2; Supplementary Table S1).

For investigating biomass compositional changes, the seasonality studies were conducted as per the specified hydrolysis method to evaluate the fluctuations in sugar level for the monthly wise-collected specimens extending from August to November to arrive at maximum concentration of reducing sugar (Fig. 2a). Overall, the obtained results showed that the total sugar as well as reducing sugar content increases linearly up to 0.242 ± 0.012 g/gdw and 0.152 ± 0.009 g/gdw during October month (post-monsoon season) with one molar acid concentration using the autoclave-assisted hydrolysis. The similar trend was also reported (Vilg et al. 2015) for the macroalgal strain, *Saccharina latissima* using thermo-chemical process, and acquired the sugar content up to 0.360 g/gdw during the optimal month of harvesting. This may be due to the changes in the environmental conditions, like temperature, pH, salinity, precipitation, wind-velocity, photoperiod, water characteristics, and nutrient concentration in the coastal ecosystem, which affects the growth attributes of macroalgae (Juneja et al. 2013; Ansari and Ghanem 2017). For example, a monthly sampling study on the Red Sea coast of Saudi Arabia found that the maximum amount of total sugar for the green macroalgal flora *Cladophora prolifera* (20.5–25.5% dw) and *Chaetomorpha linum* (20.3–23.41% dw) was obtained in the October month with varying water characteristics and maximum photosynthetic as well as adjacent pigment accumulation (Ansari and Ghanem 2017). This can be inferred from the variation in pH (8.1–8.5), temperature (5–17°C), turbidity (10–12 NTU) dissolved O_2_ concentration (4.8–6.5 mg/L), nitrogen (1.8–4.1% dw), phosphorous (0.2–0.3% dw), and potassium (1.5–1.7% dw) content, respectively, with regard to low-to-high tide condition (Ansari and Ghanem 2017). Of which, during the low tide, the green macroalgae get submerged and affected by the physicochemical parameters of seawater (e.g., pH and nutrient concentration) and other biotic factors. While in case of high tide, the green macroalgae are exposed to sunlight and atmospheric temperature that by the means of photosynthetic pigments (e.g., chlorophyll a and b) converts solar energy into carbohydrates via photosynthesis. Therefore, in the present study, the observed optimal sugar profile for the October-month isolate (MITM10) could be because of prolonged exposure to sunlight during post-monsoon season that enriched the carbohydrate composition, which was utilized as an energy resource for the growth and proliferation of *U. prolifera*.

Apart from it, the green macroalgae are known to contain structural as well as reserve polysaccharides, like cellulose and starch (25–50% of dry biomass) and additional sulphated polysaccharide as ulvan (8–29% of dry biomass) in its composition (Cesário et al. 2018). Among them, the cellulose and starch serves as the main source of carbon that give rise to neutral monomeric sugar (glucose) on hydrolysis, whereas the ulvan breaks down to acidic water-soluble sugars, such as uronic acids, sulphated L-rhamnose, xylose, and glucose (Cesário et al. 2018). Therefore, the variation accounted for the biomass sugar profile might be due to deviations in cellulose and ulvan composition with regard to seasons that varied the sugar content periodically due to desiccation stress with exposure to the tides. Thus, the rising trend for sugar content in the *U. prolifera* biomass was observed from August to October month followed by a sharp dip in November month at the end of the post-monsoon season. Alongside, the variability of the total sugar to monomeric sugar content has also been described for the various strains of macroalgae (Jang et al. 2012); however, in our sampling, the conversion ratio within two seasons for the same strain was found to be less (32.1–63%) as compared to the other macroalgal species (Jang et al. 2012). Furthermore, the presence of fermentation inhibitors (furfural and 5-hydroxy-methyl furfural) in the treated hydrolysate (resultant of 1M acid treatment) of the optimal macroalgal biomass (MITM10) was also analyzed using chloroform extraction based UV-spectrophotometry and determined to be within the reported range (0.02–0.03 g/L) for the *Ulva* species (Melo et al. 2016; Hessami et al. 2018). Hence, the fermentable monomeric reducing sugar content comprises a major proportion of the total sugar pool of the collected biomass, as profiled in Fig. 2a.

In addition, the proportion of cell wall polysaccharides also vary significantly with respect to seasonality, which subsequently affects the bioethanol production (Adams et al. 2011). For example, in the tank cultivation of *Ulva* sp., a variance in total carbohydrate pool (glucan fraction) was observed with respect to monthly variation by reaching its maxima up to 54% on dry mass basis during winter season and bioethanol yield of about 0.3g/g reducing sugar (Qarri and Israel 2020). Consequently, in this study, the fermentation efficiency of the *U. prolifera* hydrolysates containing varying sugar contents were measured for the specimens of 4 months that falls between the two different seasons (Fig. 2a and 2b). Of which, the October-month isolate (MITM10) yielded the maximum bioethanol concentration up to 6.275 ± 0.161 g/L (Fig. 2b) with theoretical bioconversion yield of 46.88% by utilizing the relative hexose sugars in comparison to other months samples (2.0–5.2 g/L) (Supplementary Table S1), as quantified by potassium dichromate method and confirmed using gas chromatography (Supplementary Figure S1). Therefore, the sugar content among the marine macroalgal taxa is an essential factor for assessing its diversity for bioethanol production. Henceforth, based on the seasonal variation for the sugar profile (Fig. 2a) ranging between 0.117–0.242 g/gdw (total sugar) and 0.037–0.152 g/gdw (reducing sugar), the highest sugar-yielding specimen of *U. prolifera* biomass (MITM10) was found to be suitable for harvesting and used for the TAH optimization studies.

### Compositional analysis

The structural components of the green macroalgal strains generally differ with reference to land plants in terms of diversity and amount of chemical constituents (C, H, O, and N). However, it shares some common features with terrestrial plants, as its microfibrillar structure is made up of β-1, 4 glycosidic linkage, and weak hydrogen bonds linking monomeric glucose molecules and other moieties in the cell wall and matrix to provide structural support. Consequently, the choice of appropriate treatment strategy for macroalgal biomass depends on its ultrastructure and biochemical composition. In this context, biochemical profiling of various green macroalgal strains has been carried out, which shows that these species differ especially with respect to total sugar, protein, and lipid composition (Ramachandra and Hebbale 2020). This relies on many ecological factors such as, seawater current, heavy metal concentration, temperature, and photoperiod. In addition, these factors also indirectly influence the elemental structure and biofunctional activities of marine macroalgae.

Hence, in order to characterize the collected biomass, the proximate analysis of the *U. prolifera* O.F. Muell. MITM10 was performed and the biochemical constituents (viz., carbohydrate, protein, lipid, and ash content) were estimated on dry weight (dw) basis as shown in Table 1. Among them, the amount of carbohydrate, protein, and lipid contents are comparable with the reported literature for the green macroalgal strains, e.g., *U. flexuosa* (Hessami et al. 2018) and *U. fasciata* (Singh et al. 2015). However, the higher ash content may be due to presence of excessive minerals, like Mn and Zn (13.92 to 304.55 μg/g dry mass of the *Donax faba*) during post-monsoon season and contaminants (e.g., ammonia and sulfur) from the effluent of the industrial port or nearby shipyard company, which inflows to seawater (Tenjing et al. 2017). *U. prolifera* O.F. Muell. MITM10 has a low-to-mid level quantity of total lipid and proteins. In addition, it contains significant amounts of total carbohydrate composition that varies with monthly collection and depends on the seasonal effect (Fig. 2a). However, in the present study, the *U. prolifera* biomass sampled in the post-monsoon season (October-month isolate) was chosen and found to contain a carbohydrate composition of 34.98 ± 3.30% dw. This variation is far lesser than the tropical *Ulva* spp., which ranged between 43 and 57% dw (Trivedi et al. 2013).

**Table 1:** Biochemical composition of *U. prolifera* O.F. Muell. MITM10

Composition	Dry weight (%) *
Carbohydrate Protein Lipid Ash	34.98 ± 3.30 12.45 ± 0.49 1.93 ± 0.07 37.83 ± 0.23

^*^Mean ± standard deviation

### Statistical optimization of TAH parameters using central composite design

The statistical methods are widely used to find the best combination of the process variables for multivariate systems with least number of experimental runs. These methods are applied for examining the relationship between the measured responses and the number of independent variables with the purpose of optimizing a method or process. In this milieu, in accordance with the earlier investigated optimization approaches (Kostas et al. 2016; Alfonsín et al. 2019; Kostas et al. 2020), the TAH parameters, such as biomass dosage, acid concentration, temperature, and hydrolysis time, showed a great impact on the reducing sugar extraction from the macroalgal biomass. Therefore, in our study, an attempt was made to enhance the RSY by optimizing the TAH process parameters (dosage, acid concentration, and hydrolysis time) under the controlled temperature (121°C) for cellulolysis as shown in Table 2. In CCD-based matrix, each row specifies the experimental conditions with the different combinations of TAH variables (A, B, and C) as individual column. The design includes the hidden replicates of the independent variables and the RSY from *U. prolifera* biomass (MITM10) was taken as the response. Inclusively, the designed matrix depicts the trend of RSY that varied between minimum and maximum value of 0.006–0.161 g/gdw for the corner as well as axial point experiments and reached its saturation at center points (0.130–0.146 g/gdw) as compared to predicted values.

**Table 2:** CCD design and its response for reducing sugar extraction

Experiment no.	TAH parameters				RSY (g/gdw)	
	(A) Dosage (% w/v)	(B) Acid concentration (M)	(C) Hydrolysis time (min)		Experimental	Predicted
1 2 3 4 5 6 7 8 9 10 11 12 13 14 15 16 17 18 19 20	(−1) 5 (+1) 15 (−1) 5 (+1) 15 (−1) 5 (+1) 15 (−1) 5 (+1) 15 (−2) 1.59 (+2) 18.41 (0) 10 (0) 10 (0) 10 (0) 10 (0) 10 (0) 10 (0) 10 (0) 10 (0) 10 (0) 10	(−1) 0.3 (−1) 0.3 (+1) 1 (+1) 1 (−1) 0.3 (−1) 0.3 (+1) 1 (+1) 1 (0) 0.65 (0) 0.65 (−2) 0.06 (+2) 1.24 (0) 0.65 (0) 0.65 (0) 0.65 (0) 0.65 (0) 0.65 (0) 0.65 (0) 0.65 (0) 0.65	(−1) 20 (−1) 20 (−1) 20 (−1) 20 (+1) 50 (+1) 50 (+1) 50 (+1) 50 (0) 35 (0) 35 (0) 35 (0) 35 (−2) 9.77 (+2) 60.23 (0) 35 (0) 35 (0) 35 (0) 35 (0) 35 (0) 35		0.085 0.061 0.126 0.124 0.099 0.065 0.134 0.147 0.150 0.104 0.006 0.128 0.097 0.161 0.130 0.147 0.136 0.130 0.136 0.146	0.083 0.046 0.120 0.120 0.100 0.067 0.150 0.150 0.140 0.110 0.014 0.120 0.110 0.150 0.140 0.140 0.140 0.140 0.140 0.140

**Fig. 3:**
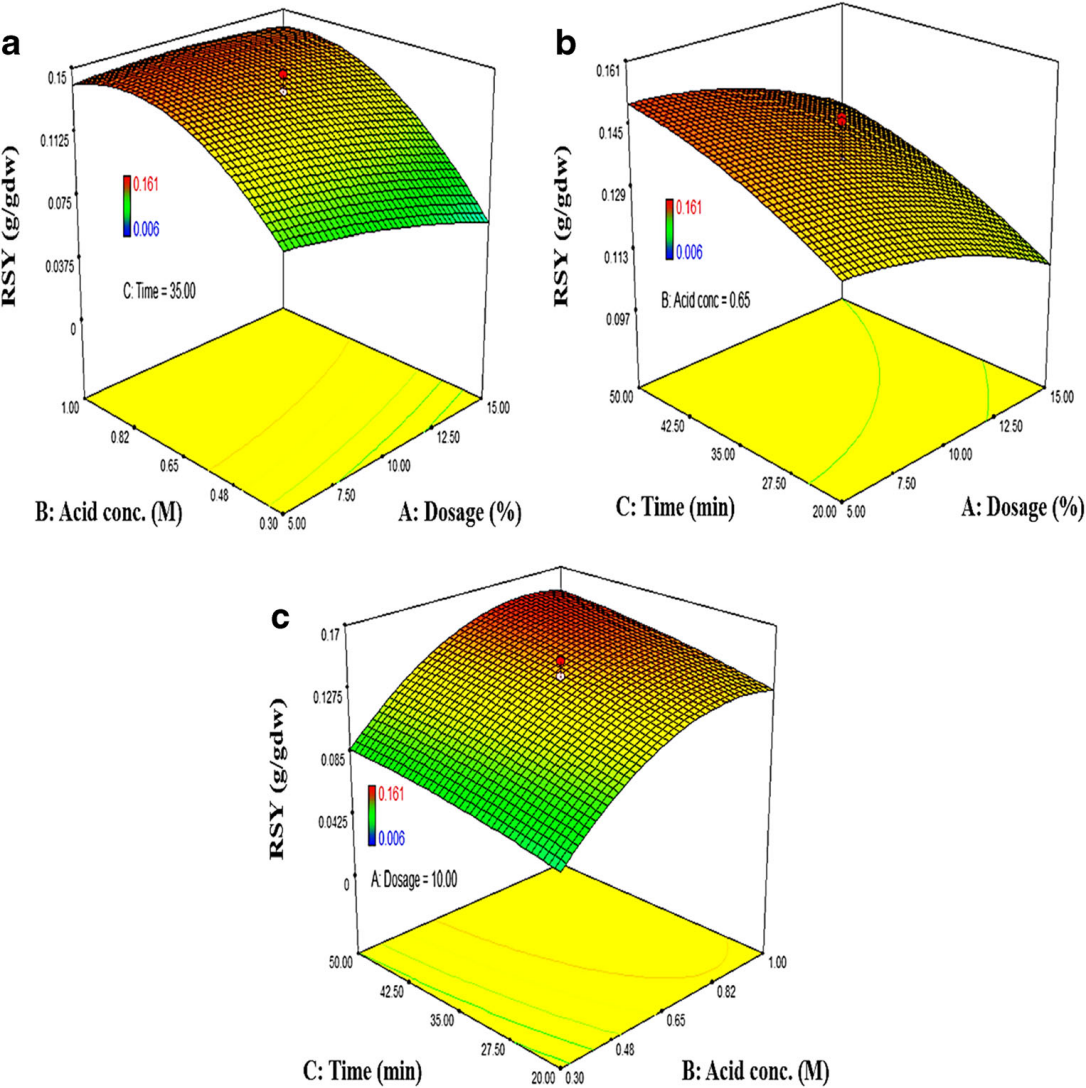
Analysis of interaction effect using response surface plots

As per CCD, different combinations of experimental runs were carried out to study the effect of TAH parameters on reducing sugar extraction (Fig. 3). The obtained results, as evident in Fig. 3 (A) with regard to first combination of TAH factors (biomass dosage and acid concentration) showed that RSY increases linearly at the constant biomass dosage (9% w/v) and hydrolysis time (35 min) with increasing sulfuric acid concentration followed by a sharp dip under the controlled process conditions. However, in the case of fixed sulfuric acid concentration (0.5 M) and constant hydrolysis time (35 min), no much variation in reducing sugar yield was observed with increasing biomass dosage. Similar trends were also reported for the green macroalgal strain, *U. intestinalis* for TAH using dilute sulfuric acid (Kim et al. 2014). This indicated that the high amount of dosage and acid concentration in TAH process does not improve the cellulolysis of macroalgal biomass. Because, the high biomass dosage tends to reduce the homogeneity of the macroalgal slurry in the TAH process due to inadequate liquid quantity and high viscosity, which is problematic to the cellulolysis reaction. Besides that, it affects the solid-to-liquid ratio between the biomass and acid, causing a decrease in RSY from macroalgae (Kim et al. 2014).

With reference to the second combination of TAH factors (biomass dosage and hydrolysis time), as shown in Fig. 3 (B), the resultant data illustrated that biomass dosage and hydrolysis time have a least interaction effect on RSY. Since the biomass dosage increases at constant hydrolysis time, no significant difference on RSY was observed. However, in terms of individual effect, a slight increase in extraction yield was achieved at the constant biomass dosage (9% w/v) and acid concentration (0.65 M) for the longer hydrolysis time. The prolonged duration (in this case, 50 min) affects the hydrolysis process which reduces cellulolysis and requires high energy consumption that indirectly increases operational cost (Tan and Lee 2015).

In the third combination of TAH factors (acid concentration and hydrolysis time), which can be deduced from Fig. 3 (C), a specific extraction pattern was observed. As in this instance, RSY rises significantly up to 0.140 g/gdw at the constant hydrolysis time (32 min) and biomass dosage (10% w/v) with increasing sulfuric acid concentration up to optimal level following a slight decrease. However, with the fixed low-mid level of acid concentration (0.5 M) and constant biomass dosage (10% w/v), RSY showed a gradual rise with hydrolysis time. This may be attributed to the longer duration of hydrolysis that allows better interaction between acid and biomass surface to favor more depolymerization of cellulose to release reducing sugar. Similar results were also reported for the green macroalgal strain, *U. lactuca* (Soliman et al. 2018). However, in some circumstances, the combination of holding time and acid concentration beyond optimal level can result in the decomposition of the monomeric sugars in the macroalgal hydrolysate due to formation of inhibitors (e.g., 2-furaldehyde and 5-hydroxymethylfurfural) (Kostas et al. 2020).

### Regression analysis

The test for significance of regression model and the results of ANOVA are given in Table 3. It represents the statistical analysis for RSY obtained from RSM-based CCD model, which indicates the sum of squares, degrees of freedom (df), mean square, Fisher’s (F) value, and probability value (*p* value) for the quadratic model and various terms.

**Table 3:** ANOVA for CCD model

Source	Sum of squares	df	Mean square	F value	*p* value Prob > F
Model	0.026	9	2.883E-003	21.19	< 0.0001
A-Dosage	1.132E-003	1	1.132E-003	8.33	0.0162
B-Acid concentration	0.013	1	0.013	97.77	< 0.0001
C-Hydrolysis time	1.796E-003	1	1.796E-003	13.21	0.0046
AB	5.951E-004	1	5.951E-004	4.37	0.0630
AC	3.125E-006	1	3.125E-006	0.023	0.8825
BC	2.113E-005	1	2.113E-005	0.16	0.7018
A^2^	2.155E-004	1	2.155E-004	1.58	0.2368
B^2^	9.065E-003	1	9.065E-003	66.64	< 0.0001
C^2^	1.439E-004	1	1.439E-004	1.06	0.3280
Residual	1.360E-003	10	1.360E-004		
Lack of fit	1.081E-003	5	2.162E-004	3.87	0.0820
Pure error	2.795E-004	5	5.590E-005		
Cor total	0.027	19			

The mean square value is the proportion of the sum of squares to corresponding df (Behera et al. 2019). Thus, the lesser mean square values (Table 3) confirmed the low variance within the experimental data. Likewise, the F value is the proportion of mean square of the model to mean square of the residual (Behera et al. 2019). Therefore, the greater F value for the model (21.19) and the parameters recommended the acceptability of the model to explain the variation in the response. Furthermore, the validity of the F value was defined by estimating the conforming *p* value to establish the statistical significance of the model terms. In analysis, the *p* value less than 0.05 indicates that the model terms are significant at 95% confidence interval. Consequently, the high *p* value of the regression model (< 0.0001), process parameters (A, B, and C), and squared effect (B^2^) determined the significance of the statistical model and parameters (Table 3). Besides, the coefficient of determination (*R*^2^) and adjusted *R*^2^ values were 0.95 and 0.90, respectively, along with non-significant value of lack-of-fit term (0.08), which showed a better fitting model with the experimental data. The coefficient of determination usually explains the variance in experimental data (Mohan et al. 2014), as it indicated that 95% of variability in RSY is attributed to the TAH variables, which confirmed the suitability of the regression model.

**Fig. 4:**
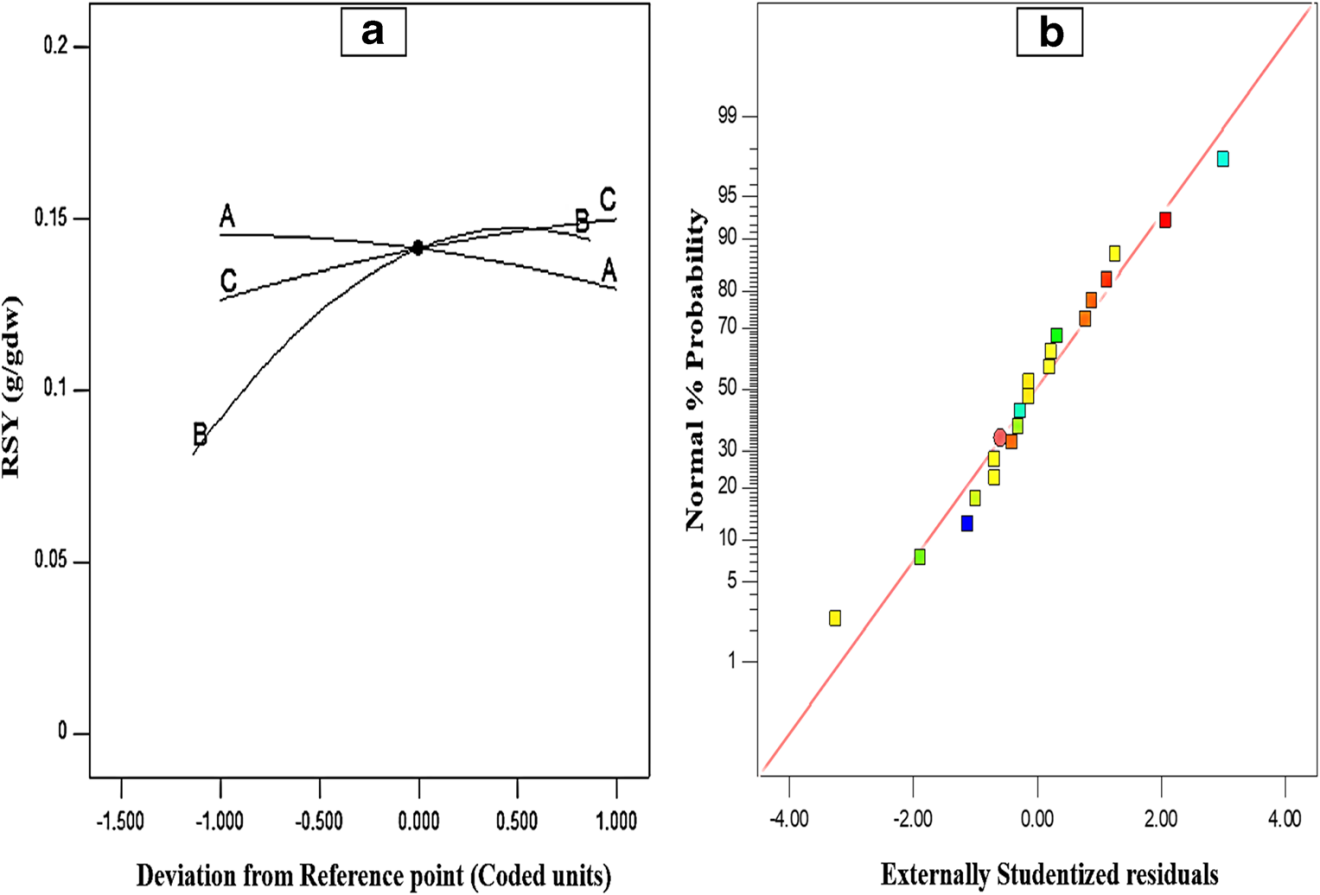
Statistical terms for determining model adequacy. (a) Perturbation plot. (b) Normal plot of experimental residuals

The model significance was also reflected with respect to coefficient of variation (CV) (Mohan et al. 2014). Generally, the CV value less than 20 is desirable. Consequently, here, the CV value of 10.09 was found to be within acceptable range, which specified the model adequacy and less deviation between the experimental and predicted values (Fig. 4). Therefore, the obtained quadratic model is reliable and can be used to predict RSY within the limits of the experimental variables for the TAH process. Furthermore, the experimental data was fitted to a polynomial equation and the mathematical relationship between the responses for RSY in terms of coded variables was determined as explained in Eq. (1).

1 $$ \mathrm{Y}=0.14-9.10\times {10}^{-3}A+0.03\ B+0.01\ C+8.62\times {10}^{-3} AB+6.25\times {10}^{-4} AC+1.62\times {10}^{-3} BC-3.86\times {10}^{-3}{A}^2-0.02\ {B}^2-3.16\times {10}^{-3}{C}^2 $$

where *Y* denotes the response for reducing sugar extraction and A, B, and C are the coded values of biomass dosage, acid concentration, and hydrolysis time, respectively. The magnitude and sign of the coefficients in the equation explain the statistical significance of the TAH parameters and their specific effects. Consequently, referring to Eq. (1), the acid concentration and hydrolysis time were found to be having a positive effect, whereas biomass dosage exerted a negative effect on the TAH process. In this context, as illustrated in Fig. 4a with the help of perturbation plot (Hamouda et al. 2015), the comparative effects of all the three TAH parameters in terms of RSY were analyzed at a particular point in the design space. As a result, it revealed the presence of low-to-sharp curvature from the center points of all the three TAH parameters, which suggested the statistical significance of each parameter (Table 3). Overall, the curvature of the TAH factors: biomass dosage (A), acid concentration (B), and hydrolysis time (C), showed that RSY rose with the increment of these factors until reaching the central point around 10% (w/v) dosage, 0.65 M H_2_SO_4_ concentration, and 35 min of hydrolysis time. Then, a sharp dip or saturation in RSY with increasing extent of the factors A, B, and C was obtained. Comparatively, the factors A and C displayed less sensitivity towards RSY, whereas the rise in RSY was observed with increment of factor B up to optimal level. Therefore, the acid concentration was determined to be the most critical parameter that influences RSY, followed by hydrolysis time and biomass dosage. In addition, the performance of the model was also analyzed by the normal probability plot for residuals (Saravanakumar et al. 2013) and percent probability of the experimental and predicted values of RSY (Fig. 4b), which showed high Pearson’s correlation coefficients (*R* = 0.975), indicating strong positive correlation between independent and dependent variables. It also presented the residual distribution and defined the difference amid experimental and predicted values of RSY to decide the quality of the model fit. As per the residual distribution, no specific trend for the predicted values of RSY was observed, which specified the model adequacy with respect to RSY within the experimental range.

### Model validation

The second-order polynomial model was obtained by fitting the experimental data to find the optimum conditions. Thereafter, experimental validation was performed in triplicate under the following conditions: dosage 11.07% (w/v), acid concentration 0.9 M, hydrolysis time 50 min. Finally, the validation run was analyzed (0.156 ± 0.005 g/gdw) and this was comparable to the predicted value (0.155 g/gdw). Overall, in this study, the amount of released reducing sugar was found to be comparable with reported literature for the macroalgal strains, *Ulva intestinalis* (Hebbale et al. 2019) and *Nemalion helminthoides* (Robin et al. 2017), where the treatment process under optimal conditions yielded the reducing sugar of about 0.135 g/gdw and 0.161 g/gdw, respectively. Thus, the obtained RSY for *U. prolifera* biomass is well within the range, which validated the efficacy of treatment method.

### Evaluation of treatment efficiency and characterization of macroalgal biomass

#### SEM analysis

**Fig. 5:**
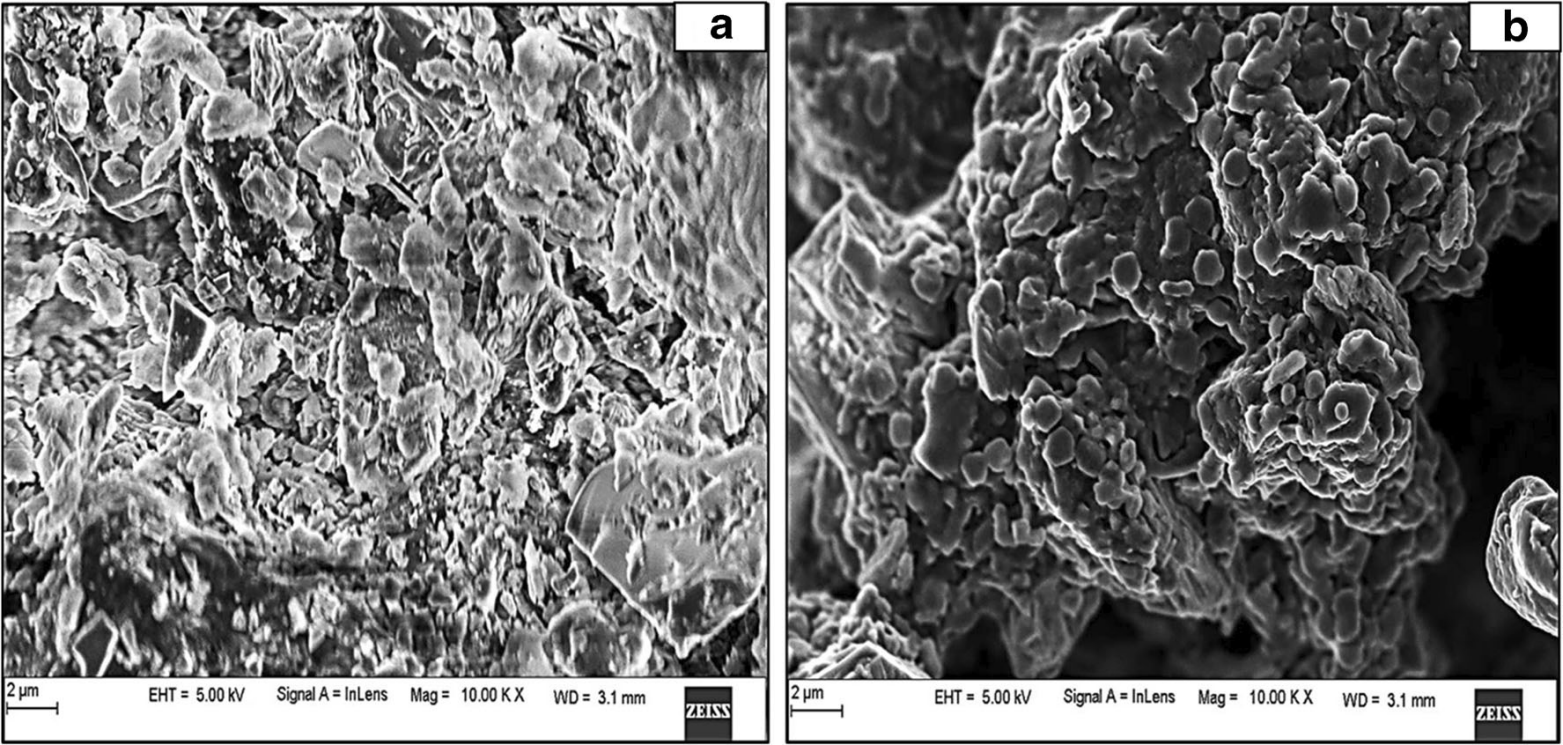
Scanning electron micrograph of biomass (**a**) before TAH process and (**b**) after TAH process

Scanning electron microscopy (SEM) was used in our study to assess the effectiveness of the TAH process with regard to structural modifications in the *U. prolifera* biomass before and after treatment (Fig. 5). The images were analyzed, which revealed the distinct morphological features in the untreated and treated macroalgal biomass, respectively. For untreated biomass, an ordered, nonporous, and rough surface with compact structure of cellulose fibers was observed without any disintegration (Fig. 5a). In contrast to this, the treated biomass presented a highly porous, loose, and smooth surface (Fig. 5b). This may be due to the high-pressure steaming that increases the surface area of biomass (Choi et al. 2013) and makes the internal cellulose fibers more accessible for acidolysis. Besides, the change in porosity suggested the solubilization of the *Ulva* polysaccharides into monomeric sugars because of its cell wall disruption using diluted sulfuric acid at 121°C. This may be ascribed to slight removal of hemicellulose fraction and liberation of the cellulose fibers from its compact structure due to shrinking effect (Hii et al. 2015).

#### FT-IR spectroscopy analysis

**Fig. 6:**
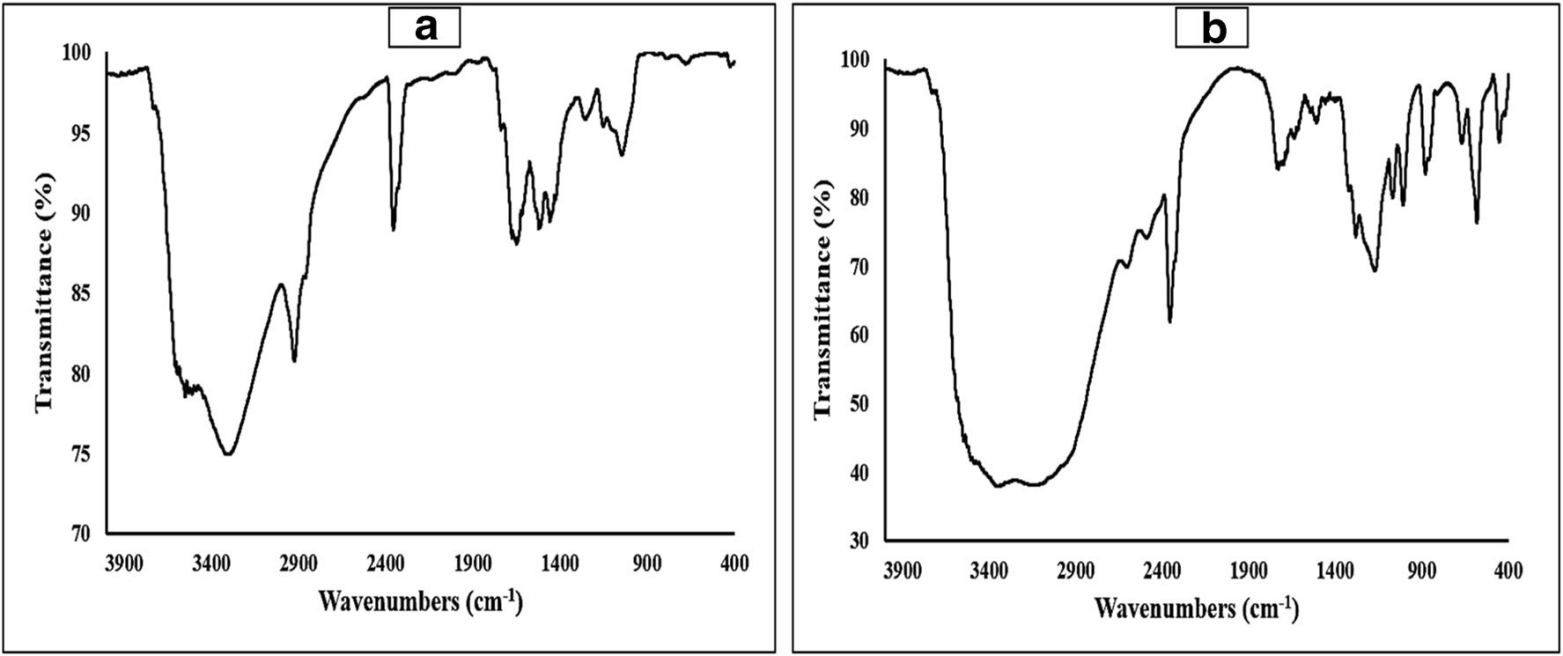
FT-IR spectra of biomass (**a**) before treatment and (**b**) after treatment

The characterization of the functional groups of macroalgal biomass was carried out using FT-IR spectroscopy. The resultant FT-IR spectra of untreated and treated *U. prolifera* biomass were analyzed, which displayed the various peaks corresponding to specific stretching and bending vibrations (Fig. 6). Among them, the typical spectrum of macroalgal biomass in terms of peak intensity and absorption maxima was studied and compared to the reported literature (Karray et al. 2015; Ceylan and Goldfarb 2015). The major functional groups were observed in the range of 3531 to 1047 cm^−1^ in the specific spectra of untreated biomass (Fig. 6a). The peak at 3531 cm^−1^ assigned to -NH stretching vibrations, 3317 cm^−1^ to -OH stretching vibrations, 2924 cm^−1^ to -CH stretching vibrations, 1651 cm^−1^ to C=O stretching vibrations, 1427 cm^−1^ to CH symmetric bending, and 1255 cm^−1^ to C-O-C as well as C-O stretching vibrations and 1047 cm^−1^ to C-O-H stretching vibrations. The spectra of the treated biomass revealed most of the peaks as it is indicated in untreated biomass with slight shift (Fig. 6b), which are -NH stretching (3487 cm^−1^), -OH stretching (3332 cm^−1^), -CH stretching (2981 cm^−1^), C=O stretching (1639 cm^−1^), CH symmetric bending (1402 cm^−1^), C-O-C and C-O stretching (1282 cm^−1^), and C-O-H stretching (1068 cm^−1^) vibrations. These changes may be due to the TAH process, which modifies the chemical composition of the macroalgal biomass, like a very broad peak in the treated biomass spectra around 3100 to 3400 cm^−1^ defines the occurrence of replaceable protons, usually from alcohol (-OH) group of monosaccharide (e.g., glucose) and carboxylic acid (-COOH) group of sugar acid (e.g., glucuronic acid). In addition, the peak at 3487 cm^−1^ specified the presence of primary or secondary amine (-NH_2_) functional group in the biomass or with the exchange of -OH group of sugars. Also, the difference in the peak intensity (post-acid treatment) at 2981 cm^−1^ (-CH stretching of aldehyde) and 1639 cm^−1^ (C=O stretching of ketone) confirmed the breakdown of cell wall to reducing sugar. Since after the TAH process, a lot of polysaccharides (holocellulose fractions) have been released, which were revealed by the bands at 1068 cm^−1^, 1172 cm^−1^, and 1282 cm^−1^, respectively (Trivedi et al. 2013), along with little amount of protein at 1639 cm^−1^ as well as lipids at 2981 cm^−1^ (Ceylan and Goldfarb 2015). Besides, the characteristic structural carbohydrate for the *Ulva* sp., i.e., ulvan, was verified in the untreated plus treated biomass by the obtained peaks at 1255 cm^−1^ (S=O stretching) and 860 cm^−1^ (C-O-S stretching) for the sulphated L-rhamnose (Trivedi et al. 2016). Thus, the structural components of the green macroalgal strain were determined and chemical alteration in the functional groups was used to verify the reducing sugar extraction from macroalgal polysaccharides.

## Conclusion

In this work, the indigenous marine macroalgal biomass (*Ulva prolifera* MITM10) was taxonomically identified, pre-processed, and screened based on seasonal trend. Thereafter, it was subjected to biochemical characterization using standard procedures and thermal acid treatment for reducing sugar extraction. The treatment was conducted according to the central composite design. Based on the regression analysis, all the three process variables (biomass dosage, acid concentration, and hydrolysis time) were found to be significant, which gave a reducing sugar yield (RSY) of about 0.156 ± 0.005 g/gdw. Thus, the reducing sugar obtained using the optimized treatment conditions may be utilized for further bioprocessing application.

## Supplementary Information

ESM 1 (DOCX 224 kb)

## Data Availability

The datasets used and/or analyzed during the current study are available from the corresponding author on reasonable request.

## Abbreviations

ANOVA: analysis of variance
CCD: central composite design
DNS: 3, 5-dinitrosalicylic acid
dw: dry weight
FT-IR: Fourier transform infrared
g/gdw: gram per gram dry weight of biomass
MITM10: Manipal Institute of Technology, collection area Malpe, sampling code 10;
RSM: response surface methodology
RSY: reducing sugar yield
SEM: scanning electron microscopy
TAH: thermal-acid hydrolysis
w/v: weight by volume
