# Supplementary material for: Evaluation of seasonal variation and the optimization of reducing sugar extraction from Ulva prolifera biomass using thermochemical method
Source: Environ Sci Pollut Res Int. 2021 Feb 5;28(42):58857–71. doi: 10.1007/s11356-021-12609-2 (PMC8541971; doi:10.1007/s11356-021-12609-2)

Table Legends

Table S1: Effect of seasonal compositional change on bioethanol production from *Ulva prolifera* biomass

Figure Legends

Figure S1: Analysis of bioethanol using gas chromatography (a) ethanol standard (b) fermentative bioethanol

Table S1:

| Season | Sampling code | Total sugar  (g/gdw) | Reducing sugar (g/gdw) | Bioethanol yield  (g/L) |
| --- | --- | --- | --- | --- |
| Monsoon | MITM8 | 0.117 ± 0.004 | 0.037 ± 0.001 | 2.023 ± 0.048 |
|  | MITM9 | 0.176 ± 0.011 | 0.094 ± 0.002 | 3.950 ± 0.271 |
| Post-monsoon | MITM10 | 0.242 ± 0.012 | 0.152 ± 0.009 | 6.275 ± 0.161 |
|  | MITM11 | 0.205 ± 0.012 | 0.129 ± 0.004 | 5.203 ± 0.121 |

Figure S1:

**
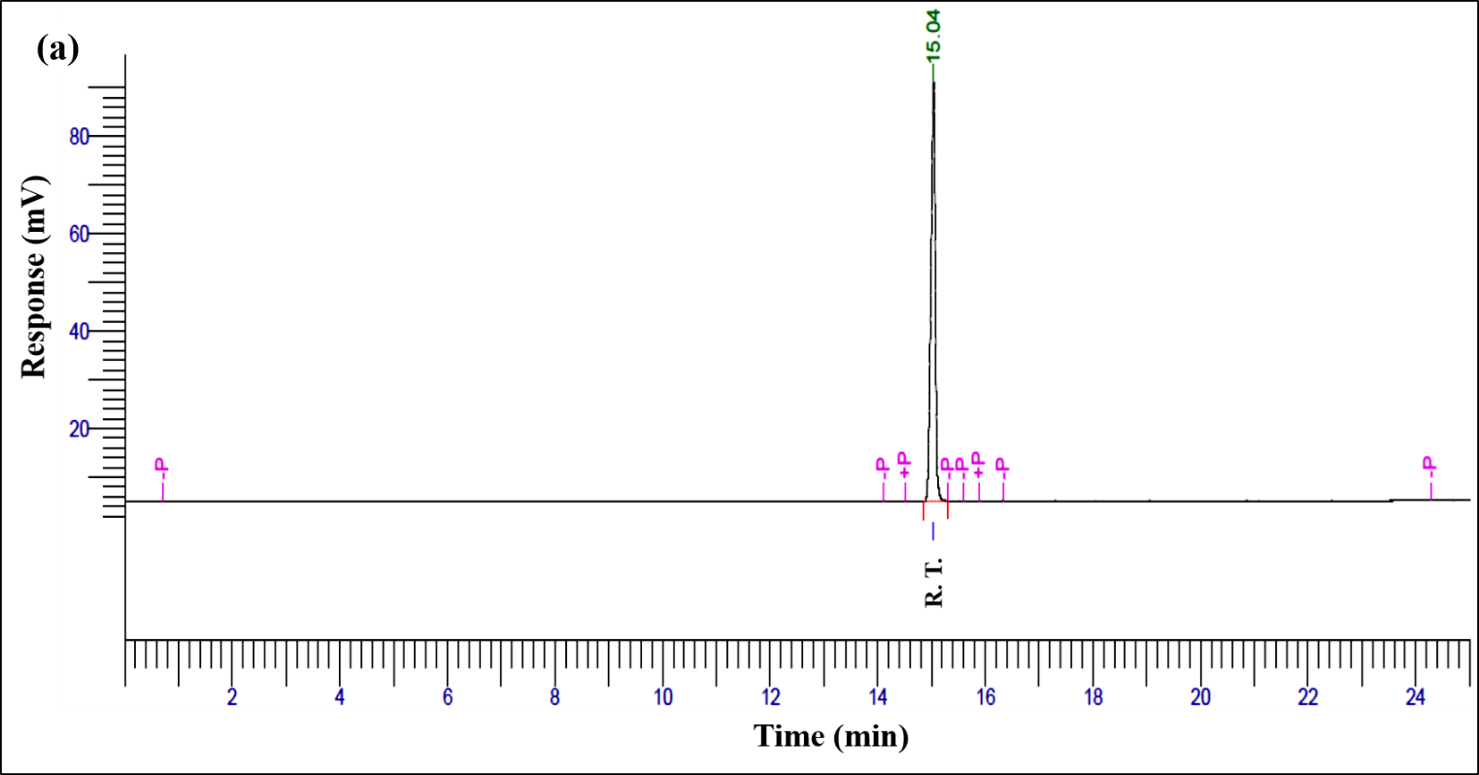
**


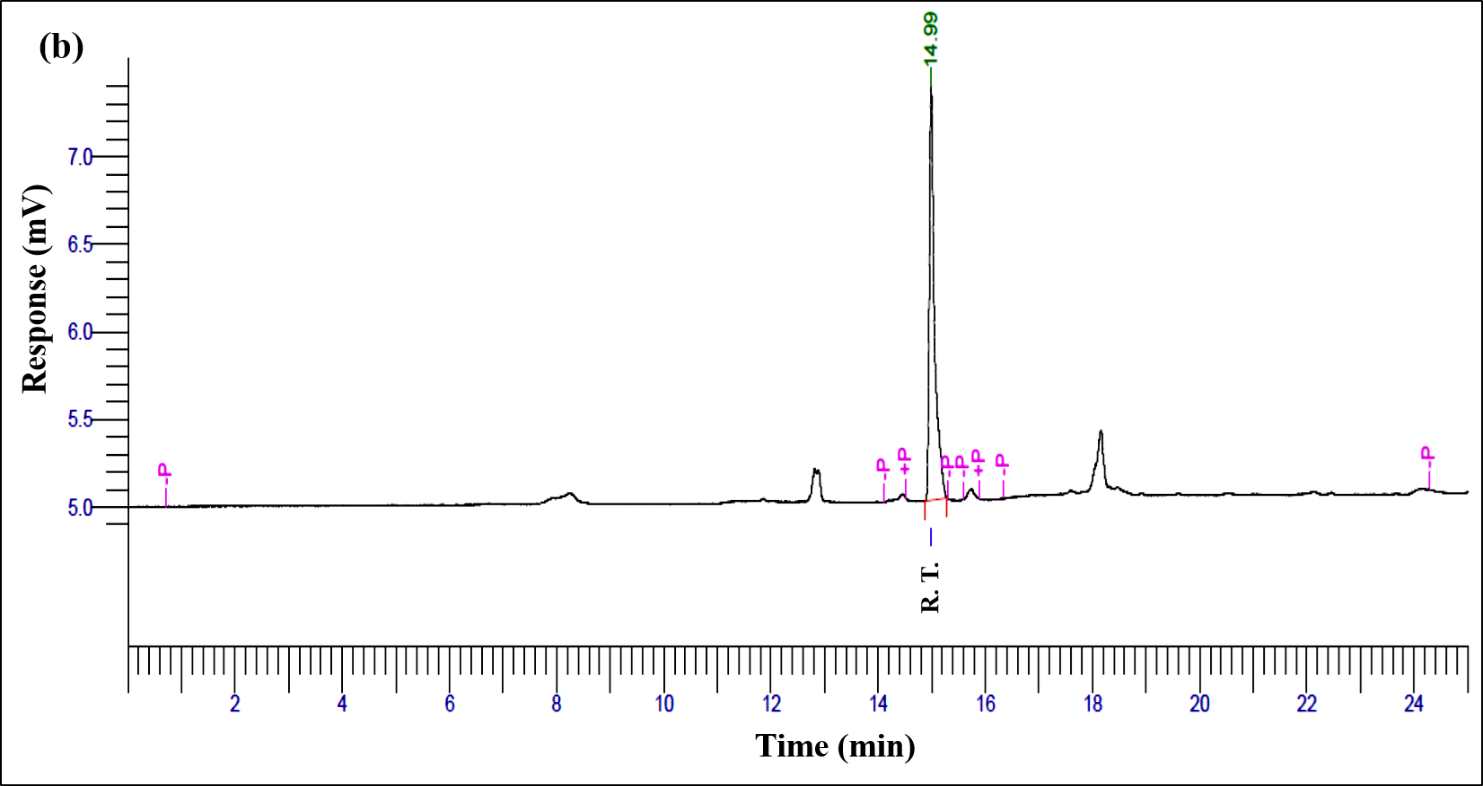

Supplement: Supplementary file 1 — (DOCX 224 kb) [file 11356_2021_12609_MOESM1_ESM.docx]
